# *Stachybotrys musae* sp. nov., *S. microsporus*, and *Memnoniella levispora* (*Stachybotryaceae*, *Hypocreales*) Found on Bananas in China and Thailand

**DOI:** 10.3390/life11040323

**Published:** 2021-04-07

**Authors:** Binu C. Samarakoon, Dhanushka N. Wanasinghe, Rungtiwa Phookamsak, Jayarama Bhat, Putarak Chomnunti, Samantha C. Karunarathna, Saisamorn Lumyong

**Affiliations:** 1 CAS Key Laboratory for Plant Biodiversity and Biogeography of East Asia (KLPB), Kunming Institute of Botany, Chinese Academy of Sciences, Kunming 650201, China; 6071105505@lamduan.mfu.ac.th (B.C.S.); wanasinghe@mail.kib.ac.cn (D.N.W.); phookamsak@mail.kib.ac.cn (R.P.); 2 Center of Excellence in Fungal Research, Mae Fah Luang University, Chiang Rai 57100, Thailand; putarak.cho@mfu.ac.th; 3 School of Science, Mae Fah Luang University, Chiang Rai 57100, Thailand; 4 World Agroforestry Centre, East and Central Asia, 132 Lanhei Road, Kunming 650201, China; 5 Centre for Mountain Futures (CMF), Kunming Institute of Botany, Kunming 650201, China; 6 Research Center of Microbial Diversity and Sustainable Utilization, Faculty of Sciences, Chiang Mai University, Chiang Mai 50200, Thailand; 7 Formerly, Department of Botany, Goa University, Goa, Res: House No. 128/1-J, Azad Co-Op Housing Society, Curca, P.O. Goa Velha 403108, India; bhatdj@gmail.com; 8 Department of Biology, Faculty of Science, Chiang Mai University, Chiang Mai 50200, Thailand; 9 Academy of Science, The Royal Society of Thailand, Bangkok 10300, Thailand

**Keywords:** new species, fungi on banana, *Musaceae*, saprobes, *Sordariomycetes*

## Abstract

A study was conducted to investigate saprobic fungal niches of *Stachybotryaceae* (Hypocreales) associated with leaves of *Musa* (banana) in China and Thailand. Three hyphomycetous taxa were collected during the dry season of 2018 and 2019. After a careful phenotypic characterization (both macro- and microscopically) and a phylogenetic tree reconstruction using a concatenated sequence dataset of internal transcribed spacer (ITS), calmodulin (*cmdA*), RNA polymerase II second largest subunit (*rpb*2), β-tubulin (*tub*2), and the translation elongation factor 1-alpha (*tef*1) gene regions, we report three species of Stachybotryaceae. *Stachybotrys musae* is introduced as a novel taxon from Yunnan, China, while *S.* *microsporus* is reported from Chiang Rai Province in Thailand on *Musa*. In addition, *Memnoniella levispora* is also reported from China for the first time.

## 1. Introduction

In the past three decades, there have been several studies on saprobic fungi associated with tropical monocotyledonous hosts [1,2,3,4,5,6,7,8,9,10]. In addition, detailed taxonomic studies have been conducted to describe and document the saprobic fungi on *Musa* across South and South East Asia [11,12,13,14,15,16].

*Stachybotryaceae* accommodates 39 genera (including *Memnoniella* and *Stachybotrys*) in *Hypocreales* [17,18]. The taxonomic histories of *Memnoniella* and *Stachybotrys* are detailed in Wang et al. [19] and Lombard et al. [20]. An updated phylogeny for *Stachybotryaceae* was provided by Lombard et al. [20] using partial 28S large sub unit (LSU), internal transcribed spacer (ITS), *rpb*2, *cmdA*, *tef*1, and *tub*2 sequence data. Previously, Smith [21] and Wang et al. [19] stated that *Memnoniella* and *Stachybotrys* are congeneric. However, Lombard et al. [20] resurrected *Memnoniella* as a distinct genus in *Stachybotryaceae*. Lin et al. [22], Doilom et al. [23], Hyde et al. [17], and Mapook et al. [24] further supported the observations of Lombard et al. [20] and treated *Memnoniella* and *Stachybotrys* as two distinct genera. Hyde et al. [17] documented nine species of *Memnoniella* with DNA sequence data. Index Fungorum [25] documented 21 names of *Memnoniella*, but ten were transferred to other genera i.e., *Brevistachys* and *Stachybotrys,* in *Stachybotryaceae* [17,24,26]. Hyde et al. [17] listed 88 species of *Stachybotrys* on the basis of Species Fungorum [27]. Currently, 30 taxa of *Stachybotrys* have DNA sequence data in GenBank.

The asexual morph of *Stachybotrys* has branched or unbranched, erect conidiophores bearing terminal, discrete, phialidic conidiogenous cells with unicellular conidia formed in chains or slimy masses [19,20,28,29]. *Memnoniella* shares a similar morphology with *Stachybotrys* [19,20,26,30] even though both genera are phylogenetically distinct. The conidia of *Memnoniella* occur on the surface as dry chains, while those in *Stachybotrys* occur as slimy masses [20]. However, much research has disregarded this dry or wet conidial disposition pattern while distinguishing *Memnoniella* and *Stachybotrys* [17,20,21,26].

*Stachybotrys* is common in soil, plant litter (hay, straw, cereal grains, and decaying plant debris), marine habitats, and air [19,20,23,24,26]. In addition, *Stachybotrys* has been detected on damp paper, cotton, linen, cellulose-based building materials (drywalls, wallpapers in indoor environments), water-damaged indoor buildings, and air ducts [5,17,19,28,31,32,33,34,35]. Most *Stachybotrys* species are cellulolytic saprobes [36], as well as plant pathogens [37,38] and endophytes [39,40,41,42,43]. *Memnoniella* species exhibit saprobic lifestyles and have been reported from dead plant materials and soil [20,39]. Some taxa of *Memnoniella* and *Stachybotrys* (*M. echinata* and *S. chartarum*) coexist in similar ecological habitats such as indoor environments [39]. Mainly, *S. chartarum* and a few other species of *Stachybotrys* (i.e., *S. elegans* and *S. microsporus*) have veterinary and medical importance as they produce several mycotoxins [44,45,46,47,48].

Many species of *Memnoniella* and *Stachybotrys* have been documented from China and Thailand. Lin et al. [23] provided a check list of *Stachybotrys* species recorded from different hosts and substrates in Thailand (*S. albipes, S. bambusicola, S. chartarum, S. elegans*, *S. microsporus, S. nephrosporus, S. palmae, S. parvisporus, S. renisporus, S. ruwenzoriensis, S. sansevieriae, S. suthepensis*, and *S. theobromae*). *Stachybotrys aksuensis* (Xinjiang), *S. biformis* (Shaanxi), *S. littoralis* (Guangdong), and *S. yushuensis* (Qinghai) were introduced from soil habitats in China [49]. In addition, *S. nielamuensis* [50] (Tibet), *S. subcylindrosporus* [33] (Hainan), *S. variabilis* [51] (Qinghai), *S. yunnanensis* [52] (Yunnan), and *S. zhangmuensis* [50] (Tibet) were described from China. *Memnoniella chromolaenae*, *M. echinata* and *M. sinensis* were reported from Yunnan Province, China and Thailand [24,39,53].

Photita et al. [11,12] and Farr and Rossman [54] documented *Stachybotrys nephrosporus*, *S. ruwenzoriensis*, and *S. theobromae* as saprobes on *Musa* from Thailand. Photita et al. [11] introduced *S. suthepensis*, which was saprobic on dead petioles of *Musa acuminata* from Chiang Mai, Thailand. In addition, *S. chartarum* [55] (Somalia) and *S. globosus* [56] (India) were found on *Musa*. *Memnoniella dichroa* (Thailand), *M. echinata* (Honduras, Japan), and *M. subsimplex* (Bermuda, Ghana, New Zealand, Sierra Leone) were also recorded on *Musa* [12,28,57,58].

Most *Stachybotrys* and a few *Memnoniella* species were introduced only on the basis of morphology [19]. The limitation of DNA sequence data in GenBank has restricted the delineation of species based on phylogeny. Wang et al. [19] and Lombard et al. [20] tried to address these research gaps and highlighted that many taxa of *Stachybotryaceae* are invalidly published. The toxicological health effects of *S. chartarum* are widely studied, but other taxa in the genus are not as well studied. Therefore, the need for a more comprehensive morpho-molecular taxonomic work on *Stachybotrys* and *Memnoniella* was recommended in recent studies [17,19,20].

We have been studying fungi associated with *Musa* [14,15,59]. The present study concentrates on saprobic *Stachybotrys* and *Memnoniella* niches on *Musa* from China and Thailand. We introduce *Stachybotrys musae* sp. nov. on *Musa* from China (Yunnan Province, Xishuangbanna), while *Memnoniella levispora* is reported from China (Yunnan) for the first time. *Stachybotrys microspores* is also reported from Chiang Rai Province, Thailand. Multi-locus phylogenetic analyses, morphological illustrations, and taxonomic discussions are provided for these taxa.

## 2. Materials and Methods

### 2.1. Sample Collection, Morphological Studies, and Isolation

Decaying leaves of an undetermined species of *Musa* with fungal structures were collected from Yunnan Province, China and Thailand during December and April of 2018 and 2019. Plant materials were transferred to the laboratory in small cardboard boxes and treated as outlined in Senanayake et al. [60].

Single-spore isolation was conducted following the methods outlined in Senanayake et al. [60]. Herbarium specimens were deposited in the Mae Fah Luang University Herbarium (Herb. MFLU), Chiang Rai, Thailand. Living cultures of each strain were deposited in the Culture Collection of Mae Fah Luang University (MFLUCC). Faces of Fungi [61] and MycoBank numbers (https://www.MycoBank.org (accessed on 18 January 2021)) were obtained for the novel taxon.

### 2.2. DNA Extraction, PCR Amplification, and Sequencing

DNA extraction, PCR amplification, and sequencing followed the methods outlined in Dissanayake et al. [62]. Five gene regions, including the internal transcribed spacer (ITS), partial calmodulin (*cmdA*), partial β-tubulin (*tub*2), translation elongation factor 1-alpha (*tef*1), and partial second largest subunit of the DNA-directed RNA polymerase II (*rpb*2), were amplified using primers ITS5/ITS4 [63], CAL-228F/CAL2Rd [64,65], Bt2a and Bt2b [66], EF1-728F/EF2 [65,67], and fRPB2-5f/fRPB2-7cR [68], respectively.

The total volume of the PCR reaction was 25 μL and consisted of 12.5 μL of 2× Power Taq PCR Master Mix (a premix and ready to use solution, including 0.1 units/μL Taq DNA Polymerase, 500 μM dNTP Mixture each (dATP, dCTP, dGTP, dTTP), 20 mM Tris-HCL pH 8.3, 100 mM KCl, 3 mM MgCl_2_, stabilizer, and enhancer), 1 μL of each primer (10pM), 2 μL of genomic DNA template, and 8.5 μL of sterilized double-distilled water (ddH_2_O). The reaction was conducted by running for 40 cycles. The annealing temperatures followed Lombard et al. [20] and Samarakoon et al. [14,59]. The amplified PCR fragments were sent to a commercial sequencing provider (TsingKe Biological Technology Co., Beijing, China). Nucleotide sequence data obtained were deposited in GenBank.

### 2.3. Sequence Alignment

Obtained sequence data were primarily checked with the Basic Local Alignment Search Tool (BLAST) in GenBank (https://blast.ncbi.nlm.nih.gov/Blast.cgi (accessed on 20 June 2020)). BLAST results and initial morphological studies revealed that our isolates belong to *Stachybotryaceae*. Other sequences used in the analyses were obtained from GenBank according to recently published papers [19,20,23] (Table 1) and BLAST search results. The single-gene alignments were made using MAFFT v. 7.036 [69] (http://mafft.cbrc.jp/alignment/server/large.html (accessed on 22 June 2020)) using the default settings and later refined where necessary using BioEdit v. 7.0.5.2 [70].

### 2.4. Phylogenetic Analyses

**Figure 1:**
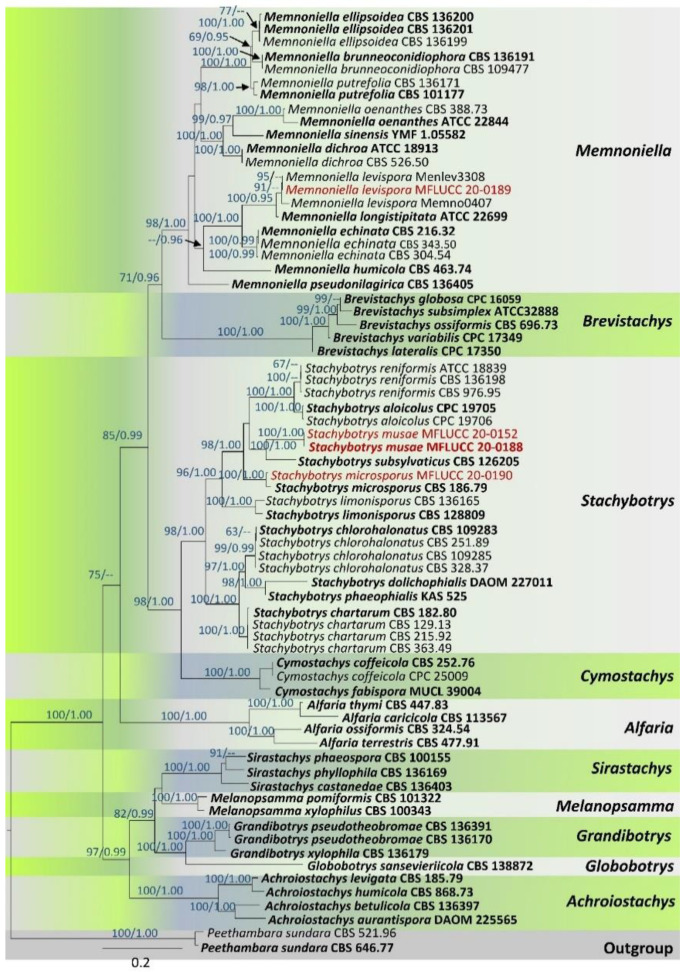
Maximum likelihood tree revealed by RAxML analyses of internal transcribed spacer (ITS), cmdA, rpb2, tub2, and tef1 sequence dataset of selected genera in Stachybotryaceae showing the phylogenetic position of Stachybotrys musae (MFLUCC 20-0152, MFLUCC 20-0188), S. microsporus (MFLUCC 20-0190), and Memnoniella levispora (MFLUCC 20-0189). Maximum likelihood bootstrap supports (≥60%) and Bayesian posterior probabilities (≥0.95 BYPP) are given above the branches, respectively. The tree is rooted with Peethambara sundara (CBS 646.77 and CBS 521.96) (Stachybotry-aceae). Strains generated in this study are indicated in red. Ex-type strains are indicated in black bold. The scale bar represents the expected number of nucleotide substitutions per site.

Maximum likelihood (ML) trees were generated using the RAxML-HPC2 on XSEDE (8.2.8) [71,72] in the CIPRES Science Gateway platform [73] using the GTR + I + G model of evolution. The latter model was selected independently for each locus of the dataset using MrModeltest v. 3.7 under the Akaike information criterion (AIC) [62]. Bootstrap supports were obtained by running 1000 pseudo-replicates. Maximum-likelihood bootstrap values equal to or greater than 60% are given above each node of the phylogenetic tree (Figure 1).

A Bayesian analysis was conducted with MrBayes v. 3.1.2 [74] to evaluate posterior probabilities (PPs) [75,76] by Markov chain Monte Carlo sampling (MCMC). Two parallel runs were conducted using the default settings but with the following adjustments: four simultaneous Markov chains were run for 2,000,000 generations, trees were sampled every 100th generation, and 20,001 trees were obtained in total. The first 4000 trees, representing the burn-in phase of the analyses, were discarded to enter the high probability region, where the states of the Markov chain are more representative of the sampling distribution. The remaining 16,001 trees were used for calculating PPs in the majority rule consensus tree. Branches with Bayesian posterior probabilities (BYPPs) equal to or greater than 0.95 are indicated above each node of the phylogenetic tree (Figure 1). The tree was visualized with the FigTree v1.4.0 program [77] and reorganized in Microsoft PowerPoint (2013).

## 3. Results

### 3.1. Phylogenetic Analyses

The combined ITS, *cmdA*, *rpb*2, *tub*2, and *tef*1 matrix comprised 70 sequences that represent selected genera in Stachybotryaceae. The best scoring RAxML tree is presented (Figure 1) with a final ML optimization likelihood value of −38,213.091. The matrix had 1833 distinct alignment patterns with 35.79% undetermined characters or gaps. Estimated base frequencies were as follows: A = 0.229585, C = 0.291579, G = 0.254548, T = 0.224288; substitution rates were as follows: AC = 1.228527, AG = 3.573013, AT = 1.331197, CG = 0.93385, CT = 5.411134, GT = 1.0; the proportion of invariable sites was I = 0.400993; the gamma distribution shape parameter was α = 1.130129. All trees (ML and BYPP) obtained from the combined ITS, *cmdA*, *rpb*2, *tub*2, and *tef*1 dataset were equal in topology and did not show any notable deviation from Lin et al. [23] and Lombard et al. [20]. Isolates of the new species, *Stachybotrys musae* (MFLUCC 20-0152 and MFLUCC 20-0188), clustered sister to *S. subsylvaticus* (CBS 12620) as a monophyletic lineage with a strong statistical support (ML = 100%, BYPP = 1.00). The new strain MFLUCC 20-0190 constituted a strongly supported monophyletic clade with *S. microsporus* (CBS 186.79) (ML = 100%, BYPP = 1.00). In addition, the new strain MFLUCC 20-0189 grouped with *Memnoniella levispora* (Menlev3308 and Memno0407) (ML = 91%, BYPP = 0.94) with moderate statistical support.

### 3.2. Taxonomy

#### 3.2.1. *Stachybotrys musae* Samarakoon & Chomnunti, sp. nov.

- MycoBank No.—MB 838529; FoF Number—FoF 09574.
- Etymology—Name reflects the host genus *Musa*, from which the novel taxon was originally isolated.
- Holotype—MFLU 20-0626.

Saprobic on dead leaves of *Musa* sp. Sexual morph: undetermined. Asexual morph: colonies on the substrate surface: effuse, usually black or blackish green. Mycelium: superficial, with light brown, septate, 5.6–7.4 μm ($\bar{x}\;$= 6.5 μm, *n =* 30) wide hyphae, sometimes forming ropes. Stroma: none. Setae and hyphopodia: absent. Conidiophores: 45–94 × 2.6–3.9 μm ($\bar{x}\;$= 71.4 × 3.2 μm, *n =* 30) macronematous, mononematous, usually unbranched, and rarely branched, often with a distinct sub-hyaline shoe-shaped base 7–9 × 3–5.7 μm ($\bar{x}\;$= 8.4 × 5.2 μm, *n =* 20). Conidiophores: usually straight or flexuous, often curved near the base, straight toward the tip, multi-septate, often with 1–7 septa, sometimes more than seven septa, hyaline or sub-hyaline at the base, pale olivaceous brown toward apex, smooth or slightly verrucose at maturity, sometimes sub-hyaline, granulate on the surface, terminating with a crown of phialides at the apex. Conidiogenous cells: monophialidic, 10–13 × 3–5 μm ($\bar{x}\;$= 11.8 × 4.4 μm, *n =* 20), discrete, in groups of 4–6 at the apex of each conidiophore, broadly fusiform, with a minute collarette at the tip. Conidia: simple, unicellular, smooth, aggregated in large, slimy, often black and glistening heads. Immature conidia: hyaline, acute at one end, rounded at the other end, spherical. Mature conidia: 5–7.5 × 4–7 μm ($\bar{x}\;$= 7.1 × 5.6 μm, *n =* 40), ellipsoidal, acute or rounded at both ends, dark brown, blackish brown or black, smooth or verrucose, sometimes covered with dark granules.

Culture characteristics—Conidia germinated on potato dextrose agar (PDA) after 48 h; germ tubes produced from germ pores. Colonies grew on PDA reaching 2 cm diameter after 3 weeks in light conditions at 25 °C, mostly immersed mycelium, slimy and minutely dense, middle of the colony orange and pinkish orange at the periphery. Radially or unevenly striated; colonies have a wrinkled appearance from the top. Conidial formation was observed only in mature cultures rarely and minutely.

Material examined—China, Yunnan Province, Xishuangbanna, on a dead leaf of *Musa* sp., 19 December 2018, D.N. Wanasinghe, BNSWN8 (MFLU 20-0626, holotype), living cultures MFLUCC 20-0188 (ex-type strain) and MFLUCC 20-0152.

**Figure 2:**
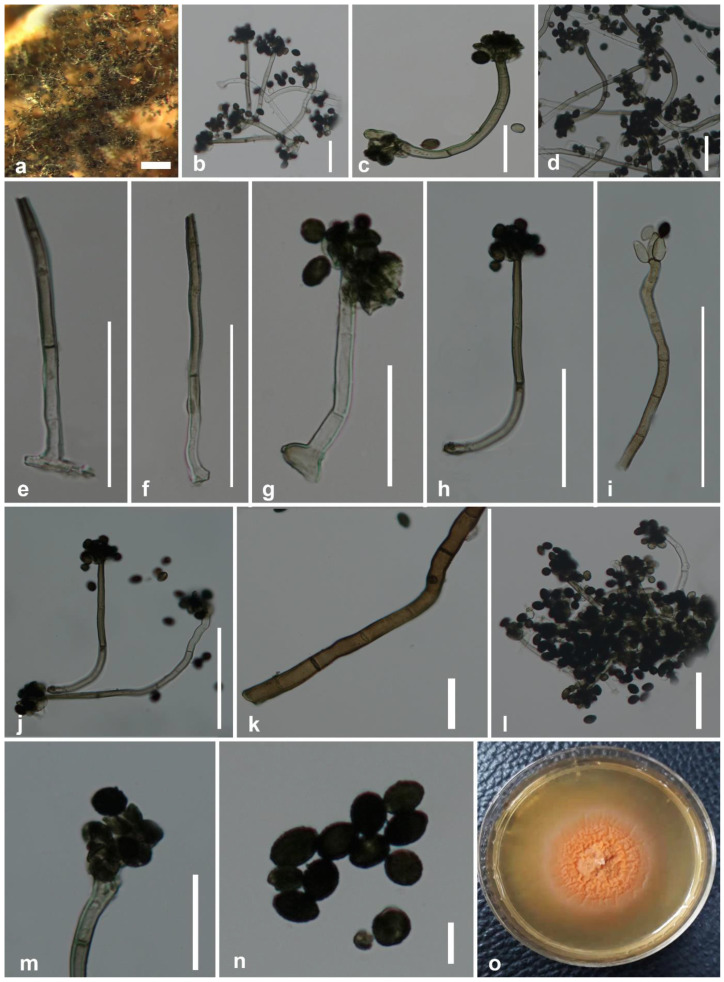
*Stachybotrys musae* (MFLU 20–0626, holotype). (**a**) Conidiophores on the substrate surface; (**b**–**d**,**g**,**h**,**j**,**m**) conidiophores with attached conidia; (**e**,**f**) conidiophores; (**i**) conidiophore with monophialidic conidiogenous cells; (**k**) mycelium; (**l**) mass of conidia and conidiophores; (**n**) conidia; (**o**) colonies on PDA after 8 weeks. Scale bars: (**a**) = 500 μm; (**j**) = 200 μm; (**c**–**i**,**l**,**m**) = 50 μm; (**b**,**f**,**g**) = 25 μm; (**n**,**k**) = 5 μm.

Notes—Based on BLASTn searches of ITS, *cmdA*, *rpb*2, and *tub*2 sequence data, *Stachybotrys musae* (Figure 2) showed a high similarity (*cmdA* = 84.34%, ITS = 94.29%, *tub*2 = 89.13%, and *rpb*2 = 90.07%) to *S. subsylvaticus* (CBS 126205). In the multigene phylogeny, *S. musae* clustered sister to *S. subsylvaticus* with ML = 100%, BYPP = 1.00 statistical support (Figure 1). Moreover, ITS sequence comparison revealed 4.94% base pair differences (without gaps) between *S. musae* and *S. subsylvaticus*. *Stachybotrys musae* (Figure 2) differs from *S. subsylvaticus* in having notably curved hyaline to olivaceous brown conidiophores, while those of *S. subsylvaticus* are straight to slightly flexuous and mostly hyaline to sub-hyaline [20]. The conidiophores of *S. subsylvaticus* are usually 1–4-septate, whereas *S. musae* has 1–7-septate or even more than 7-septate conidiophores. In addition, *S. musae* has distinct sub-hyaline shoe-shaped conidiophore bases that are absent in *S. subsylvaticus.* The apex of the phialidic conidiogenous cells of *S. subsylvaticus* is sub-hyaline to pale olivaceous brown, while *S. musae* has completely hyaline phialides. When considering the culture characteristics, the colonies on PDA of *S. subsylvaticus* are buff to pale luteous, whereas *S. musae* produces characteristic pinkish orange colonies on PDA. In our multigene analysis, *S. musae* has a close phylogenetic affinity to *S. aloicolus* and *S. reniformis.* However, *S. aloicolus* has allantoid to fusiform conidia containing 1–2 oil droplets [78]. *Stachybotrys reniformis* bears tuberculate and often globose conidia [19]. These specific features are absent in *S. musae.* Based on distinct morphological characteristics and significant statistical support from our molecular phylogenetic studies, *S. musae* is introduced herein as a new species on *Musa* from Xishuangbanna, Yunnan Province, China.

#### 3.2.2. *Stachybotrys microsporus* (B.L. Mathur & Sankhla) S.C. Jong & E.E. Davis

Saprobic on dead leaf petiole of *Musa* sp. Sexual morph: undetermined. Asexual morph: hyphomycetous. Colonies on the substrate surface are black and hairy. Conidiophores: macronematous, mononematous, often simple, erect, straight or mostly flexuous, irregularly or sympodially branched, 20–50 × 1.3–3.1 μm ($\bar{x}\;$= 32.4 × 2 μm, *n* = 20) at the base, tapering to 0.6–1.4 μm wide ($\bar{x}\;$= 0.94 μm, *n* = 20) near the apex, smooth, thick-walled, septate, hyaline at base, olivaceous brown at apex, bearing a crown of phialides at the tip. Conidiogenous cells: 3.7–7.1 × 2.5–3.1 μm ($\bar{x}\;$= 5.3 × 2.8 μm, *n* = 20), monophialidic, discrete, determinate, terminal, obovoid, with peripheral ones somewhat curved, smooth, sub-hyaline. Conidia: 7.7–14.2 × 5.1–9.8 μm ($\bar{x}\;$= 9.3 × 7.5 μm, *n* = 40) unicellular, simple, often aggregated as large glistening heads in black, when young elliptical, rounded at both ends, becoming globose, and often having pointed ends at maturity, roughened at surface, dark brown to black.

Culture characteristics—Conidia germinated on PDA after 36 to 48 h. Colonies grew on PDA reaching 2–2.5 cm diameter after 3 weeks in light conditions at 25 °C; slow-growing, flat, sparse, mycelium is completely immersed, pink, radially striated or wrinkled. Sporulation was not observed in cultures.

Material examined—Thailand, Chiang Rai Province, Mae Sai District, on dead leaf petiole of *Musa* sp., 20 April 2019, B. C. Samarakoon, BNS 30 (MFLU 20-0628), living culture MFLUCC 20-0190.

Substrates and known distribution—Soil (China and India), on *Arachis hypogaea* (Nigeria), decaying wood and sub shrubs (karst areas in Thailand), *Solanum lycopersicum* (Canada) [19,20,23,79].

**Figure 3:**
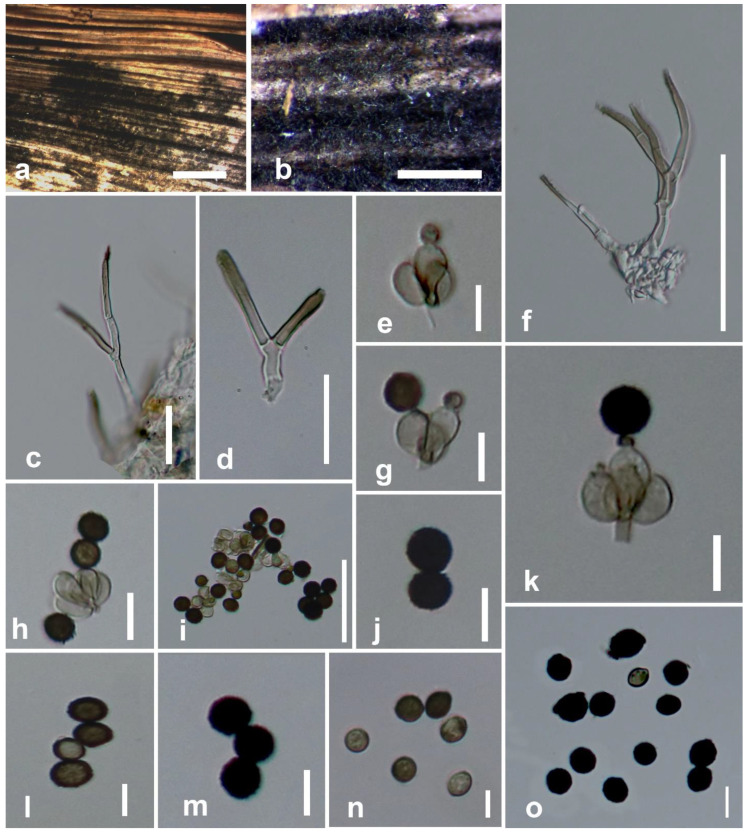
*Stachybotrys microsporus* (MFLU 20–0628). (**a**,**b**) Conidiophores on the substrate surface; (**c**,**d**,**f**) conidiophores; (**e**,**g**–**i**,**k**) conidiogenous cells with attached conidia; (**j**,**l**–**o**) conidia. Scale bars: (**a**,**b**) = 500 μm; (**f**) = 30 μm; (**i**) = 50 μm; (**e**,**g**,**h**) = 20 μm; (**c**,**d**,**j**–**m**) = 15 μm; (**n**,**o**) = 10 μm.

Notes—*Stachybotrys microsporus* (strain MFLUCC 20-0190) grouped with *S. microsporus* (strain CBS 186.79) with strong statistical support (Figure 1). All strains of *S. microsporus* described in Wang et al. [19] and Lin et al. [23] have a similar morphology (i.e., hyaline, sympodially or irregularly branched conidiophores with tapering apices) with our collection (MFLU 20-0628) (Figure 3). On the basis of DNA sequence data of a Brazil collection, Santos [80] reported that *S. globosus* is conspecific with *S. microsporus*. However, *S. globosus* was described from India, and neither an ex-type strain nor an epitype strain exists for this species. It is recommended to obtain DNA from the holotype or the ex-type of *S. globosus* to validate the conspecificity with *S. microsporus.* Previously, *S. globosus* was documented on *Musa* from India without molecular justifications [56]. Hence, in this study, we report *S. microsporus* on *Musa* from Thailand with morphological evidences and DNA sequence data.

#### 3.2.3. Memnoniella levispora Subram

Saprobic on dead leaf petiole of *Musa* sp. Sexual morph: undetermined. Asexual morph: colonies on the substrate surface, gregarious, scattered, superficial, black, powdery and bouquet-like. Conidiophores: 43.6–60 × 2.5–4.7 μm ($\bar{x}\;$= 48.7 × 3.7 μm, *n* = 20) at the base, 5–7 μm wide at swollen apex, straight or flexuous, macronematous, unbranched, bearing a crown of phialides at the apex, minutely verrucose at base, often covered in part with dark granules to black olivaceous at lower half, thick-walled, 1–3-septate. Conidiogenous cells: 4–6.9 × 2.3–3.1 μm ($\bar{x}$ = 5.8 × 2.6 μm, *n* = 20) phialidic, sub-hyaline, short and narrow at apex, clavate, ampulliform, cylindrical or broadly fusiform, without collarettes. Conidial heads: arising from conidiogenous cells, convex, round at apex and flat at base, black. Conidia: 2.5–4.3 × 1.5–3.6 μm ($\bar{x}$ = 3.5 × 2.2 μm, *n*= 20), in unbranched chains, simple, spherical to subspherical, often flattened in a plane or hemispherical, gray, dark brown to black and smooth.

Culture characteristics—Conidia germinated on PDA after 24 h. Germ tubes were produced from germ pores. Colonies grew on PDA reaching 16–21 mm diameter after 3 weeks in light conditions at 25 °C, slow-growing, crenated, flat or effuse, moderately fluffy, medium sparse, aerial, white from above, pale yellowish from below.

Material examined—China, Yunnan Province, Xishuangbanna, on dead leaf of *Musa* sp., 18 December 2018, D.N. Wanasinghe, BNSWN6 (MFLU 20-0627), living culture MFLUCC 20-0189.

Substrates and known distribution—on *Morus* (India), *Oryza sativa* (Cuba), *Roystonea regia* (Cuba), *Sanchezia* (India, Pakistan), *Tectona grandis* (Thailand) [19,22,28,81,82].

**Figure 4:**
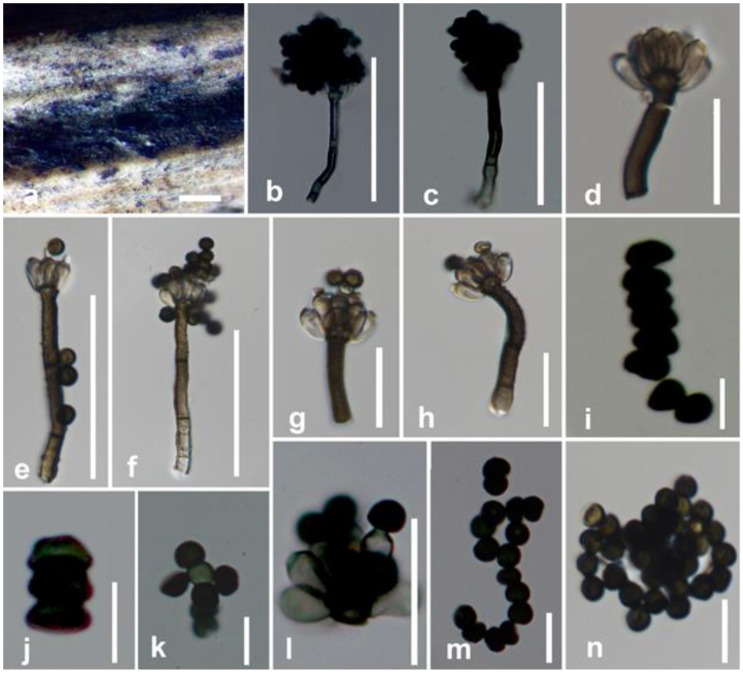
*Memnoniella levispora* (MFLU 20–0627). (**a**) Conidiophores on the substrate surface; (**b**–**g**) conidiophores and conidia; (**h**,**l**) conidiogenous cells and conidia; (**i**–**k**,**m**,**n**) conidia. Scale bars: (**a**) = 500 μm; (**b**,**c**,**e**,**f**) = 50 μm; (**d**,**g**–**l**) = 20 μm; (**i**–**k**,**m**,**n**) = 10 μm.

Notes— Our strain, MFLUCC 20-0189, grouped with strains identified as *Memnoniella levispora* (Menlev3308 and Memno0407) in GenBank with moderate statistical support (ML = 91%, BYPP = 0.94) (Figure 1). The morphological descriptions of *M. levispora* given in Wang et al. [19] and Doilom et al. [22] share similar features such as the bouquet-like fungal colonies and catenate, numerous conidia, with our strain (Figure 4). *Memnoniella levispora* was documented on *Musa* sp. from India by Munjal and Kapoor [83] using only morphological data. We report *M. levispora* as a saprobe on *Musa* sp. for the first time from Yunnan, China as a new geographical record based on morpho-molecular data. We observed that molecular data available in GenBank represent neither an ex-type strain nor an epitype strain of *M. levispora.* Hence, we highly recommend re-examining the Indian holotype to see the possibility of sequencing or epitypify the species with a new collection.

## 4. Discussion

Taxonomic evidence for the new species is further strengthened by a comparison of *Stachybotrys* taxa previously described from *Musa* based only on morphology. *Stachybotrys suthepensis* was described from a dead petiole of *Musa acuminata* by Photita et al. [11]. However, *S. suthepensis* differs from *S. musae* in having significantly verruculose, ellipsoid to cylindrical conidia which are rounded at the ends. Conidia of our new collection are not verruculose and ellipsoidal in shape with acute ends. In addition, the conidiophores of *S. musae* are notably curved compared to those formed by *S. suthepensis.* Molecular data of *S. suthepensis* are not available in GenBank for a comparison with our strain.

*Stachybotrys chartarum, S. kampalensis, S. nephrosporus,* and *S. theobromae* are distinct from the new species according to morpho-molecular data. *Stachybotrys ruwenzoriensis*, for which no DNA sequence data are available in Genbank, differs in having obovoid phialides and notably verrucose, globose to subglobose conidia. *Stachybotrys yunnanensis* was recorded from the same geographical region (Yunnan, Yunnan Province, China) as *S. musae* but differs in both morphology and phylogeny.

*Stachybotrys bambusicola* differs from the new species in having pink conidia [84]. In *S. longisporus* [20], the distinct conidiophore base is globular shaped, whereas, in *S. musae*, it is shoe-shaped. The conidiogenous cells of *S. longispora* do not have collarettes compared with those of *S. musae*. The conidiophore base of *S. nephrodes* [85] is similar to *S. musae*, but the conidial shape is different from our new species in being reniform. *Stachybotrys reniverrucosa* [35] also has notably curved conidiophores like *S. musae*, but both species can be easily differentiated by the conidial shape.

Many *Stachybotrys* taxa lack ex-type strains, and holotypes are often difficult to locate. Sequence data for several species are lacking in GenBank. Some species were established, described, and identified solely using ITS sequence data. However, constructing phylogenies only based on ITS data will not result in good tree topologies in *Stachybotrys*. Multiple sequence alignments combined with protein-coding regions result in well-resolved phylogenies with well-separated clades for *Memnoniella* and *Stachybotrys* (Figure 1). We noted the lack of other protein-coding gene regions (i.e., *cmdA*, *rpb*2, *tub*2, and *tef*1) in GenBank for many extant species of *Stachybotrys*. Differentiating *Memnoniella* and *Stachybotrys* has been problematic for over 50 years, and it was finally resolved by Lombard et al. [20]. Several genera in *Stachybotryaceae* are similar in morphology but have different molecular data [20]. Therefore, further taxa of *Stachybotryaceae* should be collected and isolated, and new sequence data should be generated for a better taxonomic resolution.

## Figures and Tables

**Table 1:** Selected taxa with their corresponding GenBank accession numbers of Stachybotryaceae used in the phylogenetic analyses. Type strains are superscripted with ^T^ and newly generated sequence data are indicated in black bold.

Taxa	Culture Collection	*cmdA*	ITS	*rpb*2	TUB2	*tef*1
*Achroiostachys aurantispora*	DAOMC 225565 ^T^	KU845784	KU845804	KU845840	NA	KU845859
*Ac. betulicola*	CBS 136397 ^T^	KU845772	KU845792	KU845831	KU845753	KU845848
*Ac. humicola*	CBS 868.73 ^T^	KU845779	KU845799	KU845837	KU845760	KU845854
*Ac. levigata*	CBS 185.79 ^T^	KU845785	KU845805	KU845841	KU845765	KU845860
*Alfaria caricicola*	CBS 113567 ^T^	KU845976	KU845983	KU846001	KU846014	KU846008
*Al. ossiformis*	CBS 324.54 ^T^	KU845977	KU845984	KU846002	KU846015	KU846009
*Al. terrestris*	CBS 477.91 ^T^	KU845979	KU845988	KU846006	KU846019	KU846011
*Al. thymi*	CBS 447.83 ^T^	KU845981	KU845990	NA	KU846021	KU846013
*Brevistachys globosa*	CBS 141056 ^T^	KU846024	KU846038	NA	KU846101	KU846085
*Br. lateralis*	CBS 141058 ^T^	KU846027	KU846043	KU846074	KU846106	KU846090
*Br. ossiformis*	CBS 696.73^T^	NA	KU846044	NA	KU846107	NA
*Br. subsimplex*	ATCC 32888 ^T^	NA	AF205439	NA	NA	NA
*Br. variabilis*	CBS 141057	KU846030	KU846047	KU846076	KU846110	KU846093
*Cymostachys coffeicola*	CPC 25009	NA	KU846053	NA	NA	NA
*Cy. coffeicola*	CBS 252.76 ^T^	KU846035	KU846052	KU846081	KU846113	KU846097
*Cy. fabispora*	CBS 136180 ^T^	KU846036	KU846054	KU846082	KU846114	KU846098
*Globobotrys sansevieriicola*	CBS 138872 ^T^	NA	KR476717	NA	KR476794	KR476793
*Grandibotrys pseudotheobromae*	CBS 136391	NA	KU846136	KU846189	KU846242	KU846215
*Gr. pseudotheobromae*	CBS 136170 ^T^	NA	KU846135	KU846188	KU846241	KU846216
*Gr. xylophilus*	CBS 136179 ^T^	KU846115	KU846137	KU846190	NA	KU846217
*Melanopsamma pomiformis*	CBS 101322 ^T^	KU846032	KU846049	KU846078	NA	NA
*Me. xylophila*	CBS 100343 ^T^	KU846034	KU846051	KU846080	NA	KU846096
*Memnoniella brunneoconidiophora*	CBS 109477	NA	KU846138	KU846192	KU846243	KU846218
*M. brunneoconidiophora*	CBS 136191 ^T^	KU846116	KU846139	KU846193	KU846244	KU846219
*M. dichroa*	CBS 526.50	KU846117	KU846140	KU846194	NA	KU846220
*M. dichroa*	ATCC 18913 ^T^	NA	AF081472	NA	NA	NA
*M. echinata*	CBS 304.54	KU846120	KU846143	KU846197	NA	NA
*M. echinata*	CBS 343.50	KU846121	KU846144	KU846198	KU846246	NA
*M. echinata*	CBS 216.32 ^T^	KU846119	KU846142	KU846196	KU846245	KU846222
*M. ellipsoidea*	CBS 136199	KU846127	KU846150	KU846204	KU846252	KU846230
*M. ellipsoidea*	CBS 136200	KU846128	KU846151	KU846205	KU846253	KU846231
*M. ellipsoidea*	CBS 136201 ^T^	KU846129	KU846152	KU846206	KU846254	KU846232
*M. humicola*	CBS 463.74 ^T^	KU846130	KU846154	KU846208	NA	KU846234
*M. levispora*	Menlev3308	NA	KF626495	NA	NA	NA
*M. levispora*	Memno0407	NA	KF626494	NA	NA	NA
***M. levispora***	**MFLUCC 20-0189**	NA	**MW477993**	NA	**MW480236**	NA
*M. longistipitata*	ATCC 22699 ^T^	NA	AF081471	NA	NA	NA
*M. oenanthes*	CBS 388.73	NA	KU846156	KU846210	NA	NA
*M. oenanthes*	ATCC 22844 ^T^	NA	AF081473	NA	NA	KU846236
*M. pseudonilagirica*	CBS 136405 ^T^	KU846132	KU846157	KU846211	KU846257	NA
*M. putrefolia*	CBS 136171	KU846133	KU846159	KU846213	KU846259	KU846238
*M. putrefolia*	CBS 101177 ^T^	NA	KU846158	KU846212	KU846258	KU846239
*M. sinensis*	YMF 1.05582 ^T^	MK772065	MK773576	MK773575	MK773574	NA
*Peethambara sundara*	CBS 521.96	NA	KU846470	KU846508	KU846550	KU846530
*Pe. sundara*	CBS 646.77 ^T^	NA	KU846471	KU846509	KU846551	KU846531
*Sirastachys castanedae*	CBS 136403^T^	KU846555	KU846660	KU846887	KU847096	KU846992
*Si. phaeospora*	CBS 100155 ^T^	KU846560	KU846666	KU846891	KU847102	KU846995
*Si. phyllophila*	CBS 136169 ^T^	KU846566	KU846672	KU846897	KU847108	KU846999
*Stachybotrys aloicolus*	CBS 137941	KU846571	KJ817889	KU846902	KJ817887	NA
*S. aloicolus*	CBS 137940 ^T^	KU846570	KJ817888	KU846901	KJ817886	NA
*S. chartarum*	CBS 129.13	NA	KM231858	KM232434	KM232127	KM231994
*S. chartarum*	CBS 215.92	NA	KU846680	KU846905	KU847116	KU847003
*S. chartarum*	CBS 363.49	NA	KU846681	KU846906	KU847117	KU847004
*S. chartarum*	CBS 182.80 ^T^	NA	KU846679	KU846904	KU847115	KU847005
*S. chlorohalonatus*	CBS 328.37	KU846619	KU846725	KU846950	KU847160	KU847048
*S. chlorohalonatus*	CBS 109283	KU846622	KU846728	KU846953	KU847163	KU847049
*S. chlorohalonatus*	CBS 251.89	KU846618	KU846724	KU846949	KU847159	KU847052
*S. chlorohalonatus*	CBS 109285 ^T^	KU846623	KU846729	KU846954	KU847164	KU847053
*S. dolichophialis*	DAOMC 227011	KU846628	KU846734	KU846958	KU847169	NA
*S. limonisporus*	CBS 136165	KU846630	KU846736	KU846960	KU847171	KU847058
*S. limonisporus*	CBS 128809 ^T^	KU846629	KU846735	KU846959	KU847170	KU847059
*S. microsporus*	CBS 186.79	KU846631	KU846737	DQ676580	KU847172	NA
*S. microsporus*	ATCC 18852 ^T^	NA	AF081475	NA	NA	NA
***S. microsporus***	**MFLUCC 20-0190**	NA	**MW477992**	NA	**MW480235**	**MW480237**
***S. musae***	**MFLUCC 20-0152**	**MW480231**	**MW477991**	**MW480229**	**MW480233**	NA
***S. musae***	**MFLUCC 20-0188^T^**	**MW480232**	**MW477990**	**MW480230**	**MW480234**	NA
*S. phaeophialis*	KAS 525 ^T^	KU846632	KU846738	KU846962	KU847173	NA
*S. reniformis*	ATCC 18839	NA	AF081476	NA	NA	NA
*S. reniformis*	CBS 136198	NA	KU846740	NA	NA	KU847063
*S. reniformis*	CBS 976.95	KU846633	KU846739	KU846963	KU847174	KU847064
*S. subsylvaticus*	CBS 126205^T^	KU846634	KU846741	KU846964	KU847175	KU847076

Abbreviations of culture collections—ATCC: American Type Culture Collection, United States of America (USA); CBS: Westerdijk Fungal Biodiversity Institute, Utrecht, the Netherlands; CPC: Working collection of Pedro Crous housed at CBS; DAOMC: Agriculture and Agri-Food Canada, Canadian Collection of Fungal Cultures, Canada; KAS: Collection of K.A. Seifert; MFLUCC: Mae Fah Luang University Culture Collection, Chiang Rai, Thailand; NA: sequence data are not available in GenBank.

## Data Availability

All sequences generated in this study are deposited in GenBank (Table 1). The finalized alignment and tree were submitted to TreeBASE (submission ID: 27607, http://www.treebase.org/ (accessed on 18 January 2021)). Morphological data are available at FigShare (https://doi.org/10.6084/m9.figshare.13602710, https://doi.org/10.6084/m9.figshare.13602719.v1, and https://doi.org/10.6084/m9.figshare.13602767 (accessed on 20 January 2021)). Specimens were deposited in the Mae Fah Luang University (MFLU) Herbarium, Chiang Rai, Thailand. Living cultures and DNA sequence data with chromatograms were deposited in the Culture Collection of Mae Fah Luang University (MFLUCC) Chiang Rai, Thailand.
